# High-throughput screening method for discovering CatSper inhibitors using membrane depolarization caused by external calcium chelation and fluorescent cell barcoding

**DOI:** 10.3389/fcell.2023.1010306

**Published:** 2023-01-19

**Authors:** Guillermina M. Luque, Liza J. Schiavi-Ehrenhaus, Martina Jabloñski, Paula A. Balestrini, Analia G. Novero, Nicolás I. Torres, Claudia E. Osycka-Salut, Alberto Darszon, Dario Krapf, Mariano G. Buffone

**Affiliations:** ^1^ Instituto de Biología y Medicina Experimental (IBYME-CONICET), Buenos Aires, Argentina; ^2^ Instituto de Biología Molecular y Celular de Rosario (CONICET-UNR), Rosario, Santa Fe, Argentina; ^3^ Instituto de Investigaciones Biotecnológicas, Universidad Nacional de San Martín (UNSAM-CONICET), Buenos Aires, Argentina; ^4^ Instituto de Biotecnología, UNAM, Cuernavaca, Mexico

**Keywords:** male contraceptive, membrane potential (EM), sperm, non-hormonal, blocker

## Abstract

The exclusive expression of CatSper in sperm and its critical role in sperm function makes this channel an attractive target for contraception. The strategy of blocking CatSper as a male, non-hormonal contraceptive has not been fully explored due to the lack of robust screening methods to discover novel and specific inhibitors. The reason for this lack of appropriate methodology is the structural and functional complexity of this channel. We have developed a high-throughput method to screen drugs with the capacity to block CatSper in mammalian sperm. The assay is based on removing external free divalent cations by chelation, inducing CatSper to efficiently conduct monovalent cations. Since Na^+^ is highly concentrated in the extracellular milieu, a sudden influx depolarizes the cell. Using CatSper1 KO sperm we demonstrated that this depolarization depends on CatSper function. A membrane potential (Em) assay was combined with fluorescent cell barcoding (FCB), enabling higher throughput flow cytometry based on unique fluorescent signatures of different sperm samples. These differentially labeled samples incubated in distinct experimental conditions can be combined into one tube for simultaneous acquisition. In this way, acquisition times are highly reduced, which is essential to perform larger screening experiments for drug discovery using live cells. Altogether, a simple strategy for assessing CatSper was validated, and this assay was used to develop a high-throughput drug screening for new CatSper blockers.

## Introduction

After ejaculation, sperm acquire the ability to fertilize in the female genital tract in a time-dependent process called capacitation (Chang, 1951; Austin, 1952). During capacitation, sperm undergo a change in the motility pattern into a more vigorous one, with a high-amplitude and asymmetric flagellar beating called hyperactivation (HA), which is critical for fertilization (Demott and Suarez, 1992; Stauss et al., 1995; Suarez and Ho, 2003). HA requires a Ca^2+^ uptake through the sperm-specific CatSper channel complex (Ren et al., 2001).

CatSper is one of the most complex ion channels characterized at the moment, both structurally and functionally. It comprises multiple subunits; the pore is formed by four homologous subunits: CatSper α 1–4 (Navarro et al., 2008; Kirichok and Lishko, 2011), that are accompanied by several auxiliary subunits: CatSper β, CatSper ɣ, CatSper δ (Liu et al., 2007; Wang et al., 2009; Chung et al., 2011), CatSper ε, CatSper ζ, EFCAB9 (Chung et al., 2017; Hwang et al., 2019) and the most recently described CatSper τ (Hwang et al., 2022; Yang et al., 2022), CatSper η, SLCO6C1 and TMEM249 (Zhao et al., 2022). Multiple testis-specific genes encode these CatSper proteins, which localize in the plasma membrane of the principal piece of mature sperm (Quill et al., 2001; Ren et al., 2001; Lobley et al., 2003; Hwang et al., 2022; Yang et al., 2022), except for CatSper ζ and EFCAB9, which are small-soluble proteins (Chung et al., 2017; Hwang et al., 2019). In addition, CatSper proteins form a unique pattern of four columns of Ca^2+^ signaling nanodomains in the plasma membrane, along the principal piece of the flagellum (Chung et al., 2014; Chung et al., 2017; Hwang et al., 2019). Sperm derived from mice lacking any of the CatSper α 1–4 genes (Ren et al., 2001; Quill et al., 2003; Qi et al., 2007) as well as human males with mutations affecting CatSper function (Avidan et al., 2003; Avenarius et al., 2009; Smith et al., 2013) are unable to hyperactivate and therefore infertile.

Furthermore, there might be species-specific adaptations of CatSper to adjust to a distinct set of stimulators within the female reproductive tract (Lishko and Mannowetz, 2018). In both rodents and primates, CatSper channels are strongly activated by intracellular alkalinization (Kirichok et al., 2006; Lishko et al., 2010), while only in primates exogenous compounds such as progesterone and prostaglandins also behave as activators (Lishko et al., 2011; Miller et al., 2015).

Since its discovery, CatSper has been considered an attractive target for male contraception because it is critical for sperm motility and is only expressed in sperm. However, the strategy of using CatSper to find a male contraceptive has not been fully implemented so far. The complex structural organization of CatSper has impeded its heterologous reconstitution and therefore the study of its regulation *in vitro*. Currently, the only accurate and specific method to evaluate CatSper function relies on patch clamp techniques. Although this approach univocally and specifically assesses CatSper function, it is time-consuming and requires sophisticated equipment and well-trained personnel. This technical limitation has hindered the development of powerful screening methods for drug discovery. However, recent advances have started to overcome this problem. For example, the rise in intracellular Ca^2+^ concentration ([Ca^2+^]_i_) after exposure to an alkaline-high K^+^ solution was evaluated as an indirect measure of CatSper function in a high-throughput drug screening (Carlson et al., 2022). Here, we propose an alternative method to specifically evaluate CatSper opening. We developed a method based on the combination of two remarkable tools: 1) the fluorescent cell barcoding (FCB) approach using flow cytometry; 2) the membrane potential (Em) assay. This powerful combination will be used to develop a high-throughput screening method for CatSper channels blockers.

FCB enables high-throughput flow cytometry increasing data robustness as well as minimizing reagent consumption (Krutzik and Nolan, 2006; Krutzik et al., 2011). This technique encodes different cell samples with a unique fluorescent signature before mixing these samples in a single tube for simultaneous data acquisition. Therefore, differences in staining volume and probes concentration that result in sample-to-sample variation are eliminated while reducing acquisition times. This enables entire 96 well plates to be run in ∼10 min. Altogether, these advantages makes FCB suitable to perform larger screening experiments for drug discovery (Krutzik et al., 2011). Up to now, this powerful technique has never been used to evaluate new drugs in mammalian sperm.

In FCB, cells labeled with unique signatures or “barcodes” of fluorescent dyes are exposed to distinct compounds from a library. The fluorescent dyes used in FCB are *N*-hydroxysuccinimide-derived, meaning reactive to amine functional groups (forming stable amide bonds) located mainly at the N-terminus and on protein lysine side chains. Cell samples with unique dye intensity distributions are obtained due to staining each of them with different concentrations of reactive fluorescent dye. Mixing differentially labeled samples into a single tube for probe staining and subsequent data acquisition is possible since the reacted dye is covalently attached to the cells enabling the non-reacted dye to be washed away. Each sample is distinguishable in the subsequent software analysis based on their fluorescence intensity in each barcoding channel (Krutzik et al., 2011).

The Em assay is based on the removal of external divalent cations by EGTA allowing CatSper to efficiently conduct monovalent cations (Kirichok et al., 2006). The magnitude of the Na^+^ influx that depolarizes the cells depends on the extent of CatSper opening (Espinosa and Darszon, 1995; Torres-Flores et al., 2011). This simple CatSper function assay has not been extensively used due to the fact that it was only validated using the non-selective pharmacological CatSper inhibitors available (Torres-Flores et al., 2011; Ernesto et al., 2015). Using CatSper1 KO sperm, we demonstrate for the first time that the depolarization induced by EGTA addition is strongly CatSper dependent.

In the present work, a simple strategy for assessing CatSper was validated, and this assay was used to develop a high-throughput drug screening for new CatSper blockers.

## Materials and methods

### Reagents

Chemicals were obtained from the following sources: bovine serum albumin (BSA) A7906 and ethylene glycol-bis (2-aminoethylether)-N,N,N,N’tetraacetic acid (EGTA) were purchased from Sigma-Aldrich (St. Louis, MO, United States), while HC-056456 from MedKoo Biosciences (Morrisville, NC, United States). Fluo-4 AM, 3,3-dipropylthiadicarbocyanine iodide (DiSC_3_(5)), CellTrace™ CFSE (C34554), CellTrace™ Violet (C34557), and pluronic acid from Invitrogen, Thermo Fisher Scientific (Waltham, MA, United States); while propidium iodide (PI) from Santa Cruz Biotechnology (Dallas, TX, United States). FDA-Approved Drug Library Mini (HY-L022M) was purchased from MedChemExpress (Monmouth Junction, NJ, United States). RU 1968 was kindly provided by Dr. Timo Strünker. HC-056456, RU 1968, Fluo-4 AM, DiSC_3_(5), CellTrace™ CFSE, CellTrace™ Violet, and pluronic acid were dissolved in DMSO; EGTA and PI were dissolved in hexa-distilled water.

### Animals

Hybrid F1 (BALB/c female × C57BL/6 male) mature (10–12 weeks-old) male mice were used for all the experiments involving FCB. CatSper1 KO (Ren et al., 2001) mice and their corresponding heterozygous (HET) siblings (C57BL/6) mature (10–12 weeks-old) male mice were also used. We have established a colony of CatSper1 KO and CatSper1 HET mice in our laboratory from founders kindly provided by Dr. Celia Santi, who obtained them from Jackson Laboratory. In one supplementary experiment, wild-type C57BL/6 mature (10–12 weeks-old) male mice were used. In all cases, mice were housed in groups of 4 or 5 in a temperature-controlled room (23°C) with lights on at 07:00 a.m. and off at 07:00 p.m. and had free access to tap water and laboratory rodent chow. All experimental procedures were carried out according to institutional animal care guidelines and were reviewed and approved by the Ethical Committees of the Instituto de Biología y Medicina Experimental, Buenos Aires #32/2021*.* Experiments were performed strictly following the Guide for Care and Use of Laboratory Animals approved by the National Institutes of Health (NIH).

### Sperm capacitation

The non-capacitating medium (NC medium) used in this study was a modified Toyoda–Yokoyama–Hosi (TYH) containing 119.3 mM NaCl, 4.7 mM KCl, 1.71 mM CaCl_2_.2H_2_O, 1.2 mM KH_2_PO_4_, 1.2 mM MgSO_4_.7H_2_O, 0.51 mM sodium pyruvate, 5.56 mM glucose, 20 mM HEPES and 10 μg/ml gentamicin (NC TYH medium). For capacitating conditions, 15 mM NaHCO_3_ and 5 mg/ml BSA were added (CAP TYH medium). In all cases, pH was adjusted to 7.4 with NaOH.

Animals were euthanized and both cauda epididymis were placed in 1 ml of NC TYH medium (without NaHCO_3_ and BSA). After 15 min of incubation at 37°C (swim-out), epididymis were removed, and sperm concentration was determined with a Neubauer chamber (1:20 dilution of swim-out sperm suspension with distilled water). Then, sperm concentration was adjusted to reach a final maximum concentration of 1 × 10^7^ cells/ml in a final volume of 100 µl of NC medium. An equal volume (100 μl) of NC or two-fold concentrated CAP medium (CAP 2X: 30 mM NaHCO_3_ and 10 mg/ml BSA) was added. Finally, sperm were incubated for 90 min at 37°C.

### Determination of [Ca^2+^]_i_ by flow cytometry

Sperm [Ca^2+^]_i_ was assessed using Fluo-4 AM as previously described (Luque et al., 2018). Briefly, after incubation in the appropriate medium, samples were washed by centrifugation, the supernatant discarded and resuspended in NC medium containing 1 μM Fluo-4 AM and 0.02% pluronic acid for 20 min at 37°C. Samples were washed again and resuspended in NC medium. Before collecting data, 3 μM of PI was added to monitor viability. Data were recorded as individual cellular events using a BD FACSCanto II TM cytometer (Biosciences; Becton, Dickinson and Company). Side-scatter area (SSC-A) and forward-scatter area (FSC-A) data were collected from 20,000 events per sample in order to define sperm population as previously described (Escoffier et al., 2012). In all cases, doublet exclusion was performed by analyzing two-dimensional dot plot FSC-A vs. forward-scatter height (FSC-H). Positive cells for Fluo-4 were collected using the filter for fluorescein isothiocyanate (FITC; 530/30), and for PI the filter for peridinin chlorophyll protein complex (PerCP; 670LP) was used. The two indicators had minimal emission overlap, but compensation was still done using single-stained controls. Data were analyzed using FlowJo software (V10.0.7).

### CatSper opening through determination of Em by flow cytometry

Sperm Em changes were assessed using DiSC_3_(5) respectively as previously described (Puga Molina et al., 2018). Briefly, after incubation in the appropriate medium, 50 nM DiSC_3_(5) was added. CatSper inhibitors (HC-056456 or RU 1968) or vehicle were used, prior dye loading, when required. In these experiments DiSC_3_(5) was not washed. Before collecting data, 3 μM of PI was added to monitor viability. Data were recorded as individual cellular events using a BD FACSCanto II TM cytometer (Biosciences; Becton, Dickinson and Company). First, basal Em was obtained after 30 s of continuous recording. Afterwards, Ca^2+^ was chelated with 3.5 mM of EGTA (pH adjusted with NaOH to ∼10 so that media pH does not change upon H^+^ release in exchange for Ca^2+^) to a value of free Ca^2+^ of 138 nM (MaxChelator) (Patton et al., 2004), and acquisition continued for additional 150 s. The sperm population was gated and doublet exclusion was performed as described above for [Ca^2+^]_i_ measurements. Positive cells for DiSC_3_(5) were collected using the filter for allophycocyanine (APC; 660/20), and for PI the filter for peridinin chlorophyll protein complex (PerCP; 670LP) was used. Compensation was done. Data were analyzed using FlowJo software (V10.0.7). Finally, DiSC_3_(5) fluorescence mean was determined in the sperm population with higher DiSC_3_(5) fluorescence before (time = 0 s) and after addition of 3.5 mM EGTA. In each condition, the fluorescence mean was normalized to the basal fluorescence (0 s).

### CatSper opening through determination of Em by spectrofluorometer

Sperm Em changes were assessed using DiSC_3_(5), as previously described (Ritagliati et al., 2018). Cells were loaded with 1 μM of the Em-sensitive dye DiSC_3_(5) for 2 min. An incubation with CatSper inhibitor (10 μM HC-056456) or vehicle was conducted, prior dye loading, when required. Sperm were transferred to a gently stirred cuvette at 37°C, and the fluorescence was monitored with a Varian Cary Eclipse fluorescence spectrophotometer at 620/670 nm excitation/emission wavelengths. CCCP (0.5 μM) was added as mitochondrial un-coupler to avoid mitochondrial contribution to recorded Em. Recordings were initiated when steady-state fluorescence was reached. Ca^2+^ was chelated with 3.5 mM of EGTA (pH adjusted with NaOH to ∼10 so that media pH does not change upon H^+^ release in exchange for Ca^2+^) to a value of free Ca^2+^ of 138 nM (MaxChelator) (Patton et al., 2004). The fluorescence change after EGTA addition was presented as ΔAFU (delta arbitrary fluorescence units), F_EGTA_ − F_R_, where F_EGTA_ represents fluorescence intensity after EGTA addition and F_R_ is the mean of 1 min of acquisition before the addition of EGTA (Torres-Flores et al., 2011; Ernesto et al., 2015). We wait for ∼1 min after EGTA addition to get a stable signal and that point is used for calculation (F_EGTA_). A normalization to the mean obtained in the control condition (CatSper1 HET or NC) was used.

### FCB protocol in combination with Em assay by flow cytometry

#### Preparation of the plate with reagents

A library of compounds was used. These drugs were provided either in DMSO or aqueous medium at a concentration of 10 mM. After the appropriate intermediate dilution (0.19 mM), a volume of 5 µl was added to each well to reach a final concentration of 10 µM.

#### Preparation of the cells

Cells were obtained by swim-out as previously described.

#### Preparation of the barcoding dyes

Two different dyes were used to produce 12 barcodes: three increasing concentrations of CellTrace™ CFSE (C34554) in combination with four increasing concentrations of CellTrace™ Violet (C34557).

In 1.5 ml microcentrifuge tubes, a three-fold serial dilution of the 5 mM stock solution of CellTrace™ CFSE (in DMSO) was performed in NC TYH medium to generate solutions at the following concentrations (3X of the final concentration that will be used to barcode the cells): CellTrace™ CFSE tube 1: 0.003 µM (final concentration 0.001 µM); CellTrace™ CFSE tube 2: 0.75 µM (final concentration 0.25 µM); CellTrace™ CFSE tube 3: 3 µM (final concentration 1 µM).

In 1.5 ml microcentrifuge tubes, a four-fold serial dilution of the 5 mM stock solution of CellTrace™ Violet (in DMSO) was performed in NC TYH medium to generate solutions at the following concentrations (3X of the final concentration that will be used to barcode the cells): CellTrace™ Violet tube 1: 0.075 µM (final concentration 0.025 µM); CellTrace™ Violet tube 2: 3 µM (final concentration 1 µM); CellTrace™ Violet tube 3: 15 µM (final concentration 5 µM); CellTrace™ Violet tube 4: 60 µM (final concentration 20 µM).

Thirty µl of each corresponding FCB dye was added to each well.

Different lots of fluorescent dyes or probes may have distinct levels of reactivity, so it is recommended that every time a new batch is opened to be tested and optimized before use.

#### Addition of samples to each barcoding dye combination

A volume of 30 µl of sperm suspension at a final concentration between 20–30 × 10^6^/ml was added to each well carefully, submerging the pipette tip in the FCB dye solution (Supplementary Figures S1A, B). Sperm concentration was determined with a Neubauer chamber (1:20 dilution of swim-out sperm suspension with distilled water). Every well contained: 30 µl sperm sample + 30 µl CellTrace™ Violet 3X + 30 µl CellTrace™ CFSE 3X + 5 µl compound (final volume 95 μl, with sperm suspension at a final concentration between 6–9 × 10^6^/ml in each well). An incubation for 20 min at 37°C in the dark was performed, making sure that the plate was well covered to avoid loss of volume by evaporation. Then, 205 µl of NC TYH medium was added to each well.

#### Combination of the barcoded samples

Immediately after, barcoded samples were combined by transferring all the volume from each well to a 15 ml tube containing additional 5 ml of warmed NC TYH medium. The total volume was 8.6 ml in the new combined tube (300 μl × 12 + 5 ml). Mouse sperm were washed by centrifugation for 15 min at 700 g at room temperature to remove the excess of unbound dyes. Immediately after centrifugation, without removing supernatant 150 µl of the pellet was transferred to a new 1.5 ml microcentrifuge tube containing 50 µl of DiSC_3_(5) 4X (200 nM, final concentration 50 nM).

#### Flow cytometry acquisition

Before collecting data, 3 μM of PI was added to monitor viability. Data were recorded as individual cellular events using a BD FACSCanto II TM cytometer (Biosciences; Becton, Dickinson and Company). Positive cells for CellTrace™ CFSE were collected using the filter for fluorescein isothiocyanate (FITC; 530/30), for CellTrace™ Violet the filter for Pacific Blue™ AmCyan (450/50), for DiSC_3_(5) the filter for allophycocyanine (APC; 660/20) and for PI the filter for peridinin chlorophyll protein complex (PerCP; 670LP) were used.

For CatSper opening assay, after 30 s of continuous recording (basal Em), Ca^2+^ was chelated with 3.5 mM of EGTA to a value of free Ca^2+^ of 138 nM (MaxChelator) (Patton et al., 2004), and acquisition continued for additional 150 s.

#### Data analysis

In all cases, doublet exclusion was performed by analyzing two-dimensional dot plot FSC-A vs. FSC-H. Compensation was done by performing the appropriate unmixed barcoded samples compensation controls in each experiment. Data were analyzed using FlowJo software (V10.0.7).

### Statistical analysis

Statistical analyses were performed using the GraphPad Prism 6 software (La Jolla, CA, United States). Data are expressed as mean ± standard error of the mean (SEM) of at least five independent experiments from different mice for all determinations. The differences between means of only two groups were analyzed using a *t*-test. Two-way analysis of variance (ANOVA) for independent measures was performed to analyze normalized median fluorescence intensity of Fluo-4 and percentage of sperm with high Fluo-4 fluorescence, for the effects of medium × genotype. Post hoc Sidak’s test was used when necessary. A probability (p) value of *p* < 0.05 was considered statistically significant. Parametric or non-parametric comparisons were used as dictated by data distribution using D’Agostino and Pearson omnibus normality test.

## Results

### The membrane potential (Em) assay is a specific and sensitive tool for analyzing CatSper function

**FIGURE 1:**
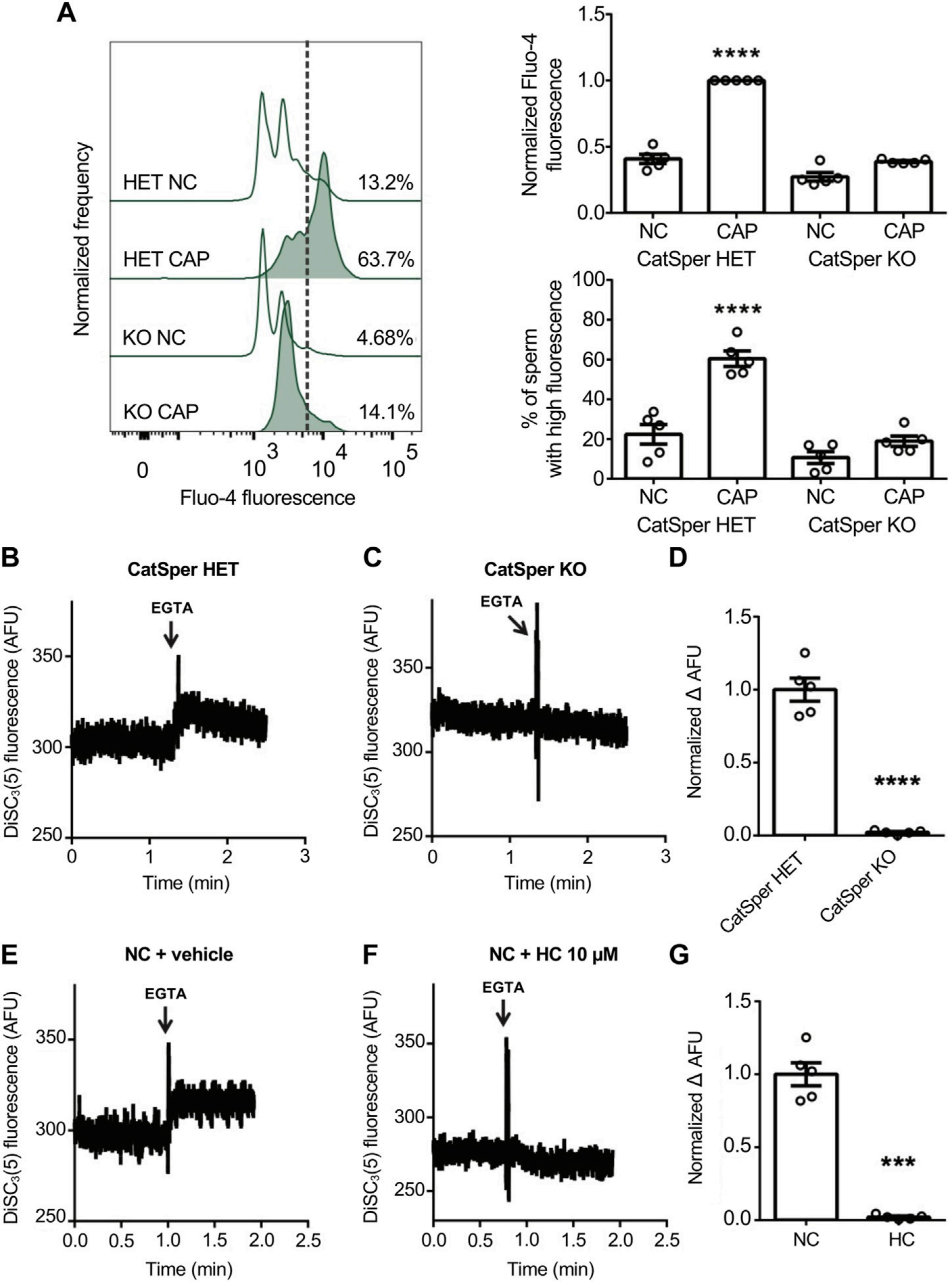
CatSper activity was analyzed by measuring Em in a population assay by spectrofluorometry, where extracellular DiSC_3_(5) fluorescence was determined. Effect of Ca^2+^ removal by EGTA 3.5 mM addition on NC sperm loaded with 1 μM DiSC_3_(5). **(A)** Sperm incubated for 90 min under capacitating conditions (CAP) or non-capacitating conditions (NC) were analyzed. Representative histograms of normalized frequency vs. Fluo-4 fluorescence of non-PI-stained sperm (live), with the corresponding percentage of sperm that increased Fluo-4 fluorescence, are shown. Normalized median fluorescence intensity, compared with the control condition (CatSper1 HET CAP), of Fluo‐4. The percentage of sperm that responds by increasing the [Ca^2+^]_i_ was established in the CAP control condition (CatSper1 HET CAP) and extrapolated to the other conditions (dashed line). Values represent the mean ± SEM of 5 independent experiments. Two‐way ANOVA showed a significant interaction (genotype × medium), P_interaction_ < 0.001. ****p < 0.0001 represents statistical significance between control (CatSper1 HET CAP condition) and all other conditions. Sidak’s multiple comparisons test was performed. **(B, C)** Representative Em recordings are shown: DiSC_3_(5) fluorescence traces (AFU: arbitrary fluorescence units) through time in CatSper1 HET **(B)** and CatSper1 KO **(C)** sperm. **(D)** Summary of normalized ∆AFU after EGTA addition (F_EGTA_) and before (resting: F_R_) compared to the mean obtained in the control condition (CatSper1 HET). Data represents the mean ± SEM of 5 independent experiments. ****p < 0.0001 represents statistical significance vs. control (CatSper1 HET). Unpaired *t*-test was performed. **(E, F)** Representative Em recordings are shown: DiSC_3_(5) fluorescence traces (AFU, arbitrary fluorescence units) through time in control (NC + vehicle) **(E)** and 10 µM HC-056456 **(F)** treated sperm. **(G)** Normalized ∆AFU after EGTA addition (F_EGTA_) and before (resting: F_R_) compared to the mean obtained in the control condition (NC + DMSO). Data represents the mean ± SEM of 5 independent experiments. ***p < 0.001 represents statistical significance vs. control (NC + DMSO). Paired *t*-test was performed.

The Em sensitive probe, DiSC_3_(5), is a positively charged carbocyanine dye that partitions into sperm cells according to their Em but independently on the nature of ionic fluxes, making it suitable for potential measurements of the plasma membrane. Em measurement with DiSC_3_(5) has long been used in mouse sperm being a robust and reproducible technique (Demarco et al., 2003; De La Vega-Beltran et al., 2012; Stival et al., 2015; Ritagliati et al., 2018; Stival et al., 2018; Luque et al., 2021). Em depolarization favors the efflux of dye out of the cell, resulting in an increase of DiSC_3_(5) extracellular fluorescence that correlates with a decrease of the intracellular one (Stam et al., 2011; Mei et al., 2015). Previous results from our and other groups employed this approach using CatSper inhibitors (Torres-Flores et al., 2011; Ernesto et al., 2015; Ritagliati et al., 2018; Stival et al., 2018). However, the ultimate validation using sperm from CatSper KO mice was not yet achieved. First, the phenotype of the mice used in the following experiment was corroborated by flow cytometry [Ca^2+^]_i_ assessment using the Ca^2+^-sensitive probe Fluo-4. Under capacitating conditions the absence of the functional CatSper channel (CatSper1 KO) displayed a significant decrease in [Ca^2+^]_i_ in comparison with the capacitation-induced rise observed in heterozygous mice (CatSper1 HET) (Figure 1A). This can be visualized by a decrease in the normalized median fluorescence intensity of Fluo-4, as well as in the percentage of sperm that displayed a rise in [Ca^2+^]_i_ (Figure 1A). Second, by measuring extracellular DiSC_3_(5) fluorescence by spectrofluorometry, it was determined that CatSper1 HET sperm displayed a Em depolarization caused by the rapid Na^+^ influx (Figures 1B, D). In contrast, sperm from CatSper1 KO mice failed to increase extracellular DiSC_3_(5) fluorescence after the addition of EGTA (Figures 1C, D).

Furthermore, consistent with previous reports using pharmacological CatSper blockers (Ritagliati et al., 2018; Stival et al., 2018), the addition of the CatSper inhibitor HC-056456 in wild-type sperm significantly decreased the magnitude of depolarization (Figures 1E–G).

### Determination of CatSper function using flow cytometry

This Em assay was adapted for flow cytometry that has the advantage of discriminating between live and dead sperm, as well as analyzing the intracellular fluorescence in individual cells.

**FIGURE 2:**
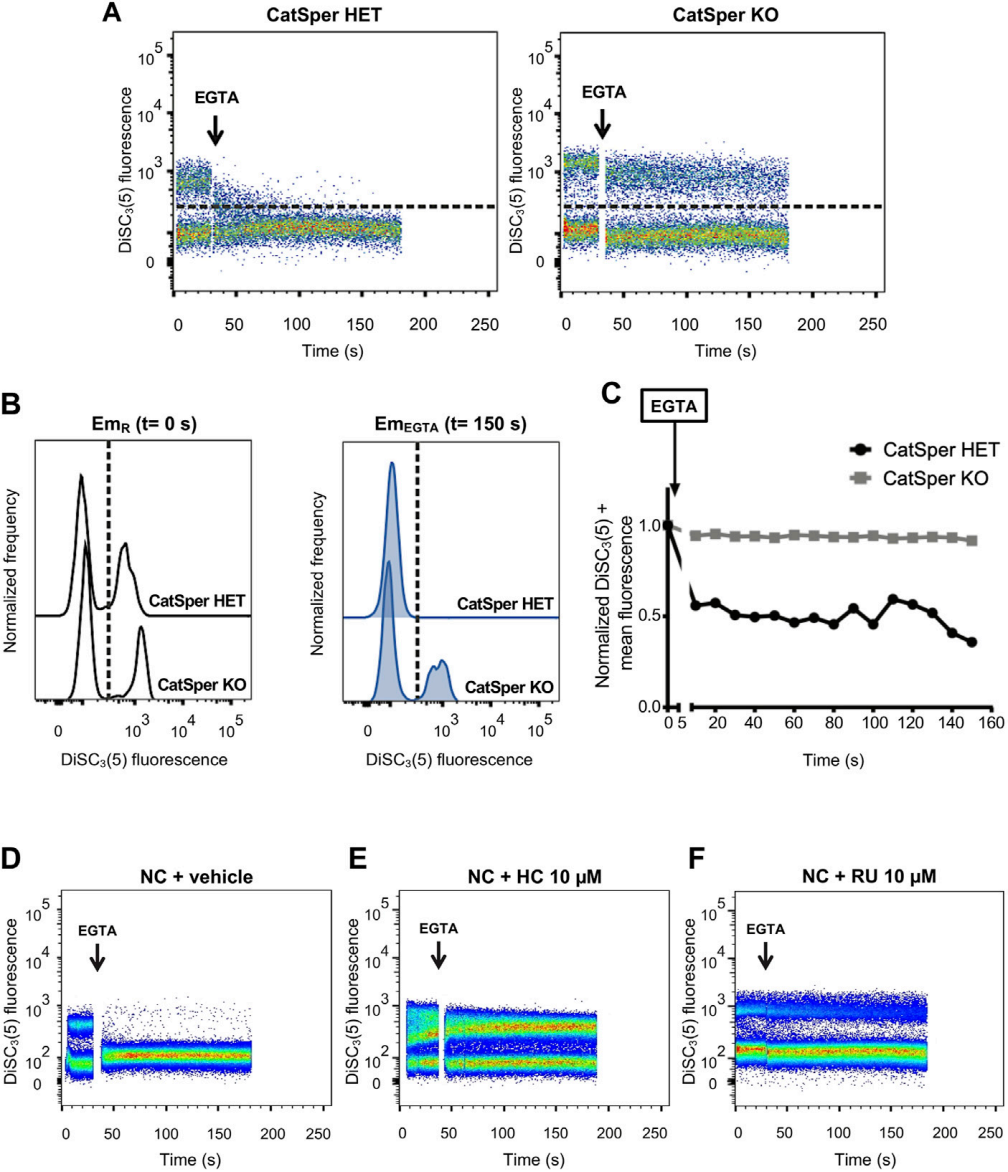
CatSper activity was assessed by measuring Em in individual live cells by flow cytometry, where intracellular DiSC_3_(5) fluorescence was determined. **(A)** Representative 2D dot plot of DiSC_3_(5) fluorescence vs. time (seconds) analysis, from 5 independent experiments, are shown. The addition of 3.5 mM EGTA is indicated using a black arrow. The population of sperm that responds by decreasing the fluorescence values was established in the CatSper1 HET control condition and used as a reference for the other conditions (dashed line). Sperm cells above the dashed line [higher DiSC_3_(5) fluorescence] were arbitrarily identified as DiSC_3_(5) +. **(B)** Histogram analysis depicting normalized frequency of sperm vs. DiSC_3_(5) fluorescence before EGTA addition (resting: Em_R_) and after (Em_EGTA_) were performed for both CatSper1 HET and CatSper1 KO samples. **(C)** DiSC_3_(5) fluorescence mean was determined in the sperm population with higher DiSC_3_(5) fluorescence: DiSC_3_(5) + [dashed line in **(A)**] before (time = 0 s) and after (black arrow) addition of 3.5 mM EGTA. In each condition, the fluorescence mean was normalized to the basal fluorescence (0 s). **(D, F)** Representative 2D dot plot of DiSC_3_(5) fluorescence vs. time (seconds) analysis in the absence or presence of pharmacological CatSper blockers. The addition of 3.5 mM EGTA (black arrow) provokes a decrease in the population of sperm with high DiSC_3_(5) fluorescence due to Em depolarization in the control condition **(D)** but not in the presence of 10 µM HC-056456 **(E)** or 10 µM RU 1968 **(F)**.

When sperm were loaded with DiSC_3_(5) and analyzed by flow cytometry, two clearly distinguishable populations were observed in basal conditions: a subset of cells with low DiSC_3_(5) fluorescence that corresponds to sperm with less hyperpolarized Em and another with higher fluorescence representing more hyperpolarized Em sperm (Figures 2A, B).

When CatSper is functionally present (CatSper1 HET), the addition of EGTA promotes that most of the cells (∼70%–90%) that displayed high DiSC_3_(5) fluorescence migrate to the low fluorescence subset as a result of the depolarization produced by Na^+^ influx upon Ca^2+^ chelation (Figures 2A–C). This is not observed when CatSper is absent (CatSper1 KO), in which a population of sperm with high DiSC_3_(5) fluorescence remained detectable after EGTA addition (Figures 2A–C). Similarly, pharmacological inhibition of CatSper using HC-056456 or RU 1968 (Rennhack et al., 2018; Curci et al., 2021) (two well characterized CatSper inhibitors), also prevented the EGTA-induced depolarization (Figures 2D–F). This pharmacological approach was also tested using two different mouse strains, C57BL/6 and hybrid F1 (BALB/c female × C57BL/6 male), and no relevant differences were found (Supplementary Figure S2). In our screening, we have chosen 10 µM HC-056456 as a positive control presented in all the experiments. As shown in Supplementary Figure S3, HC-056456 inhibits CatSper in a concentration-dependent manner.

### Fluorescent cell barcoding (FCB) flow cytometry in live mouse sperm

**FIGURE 3:**
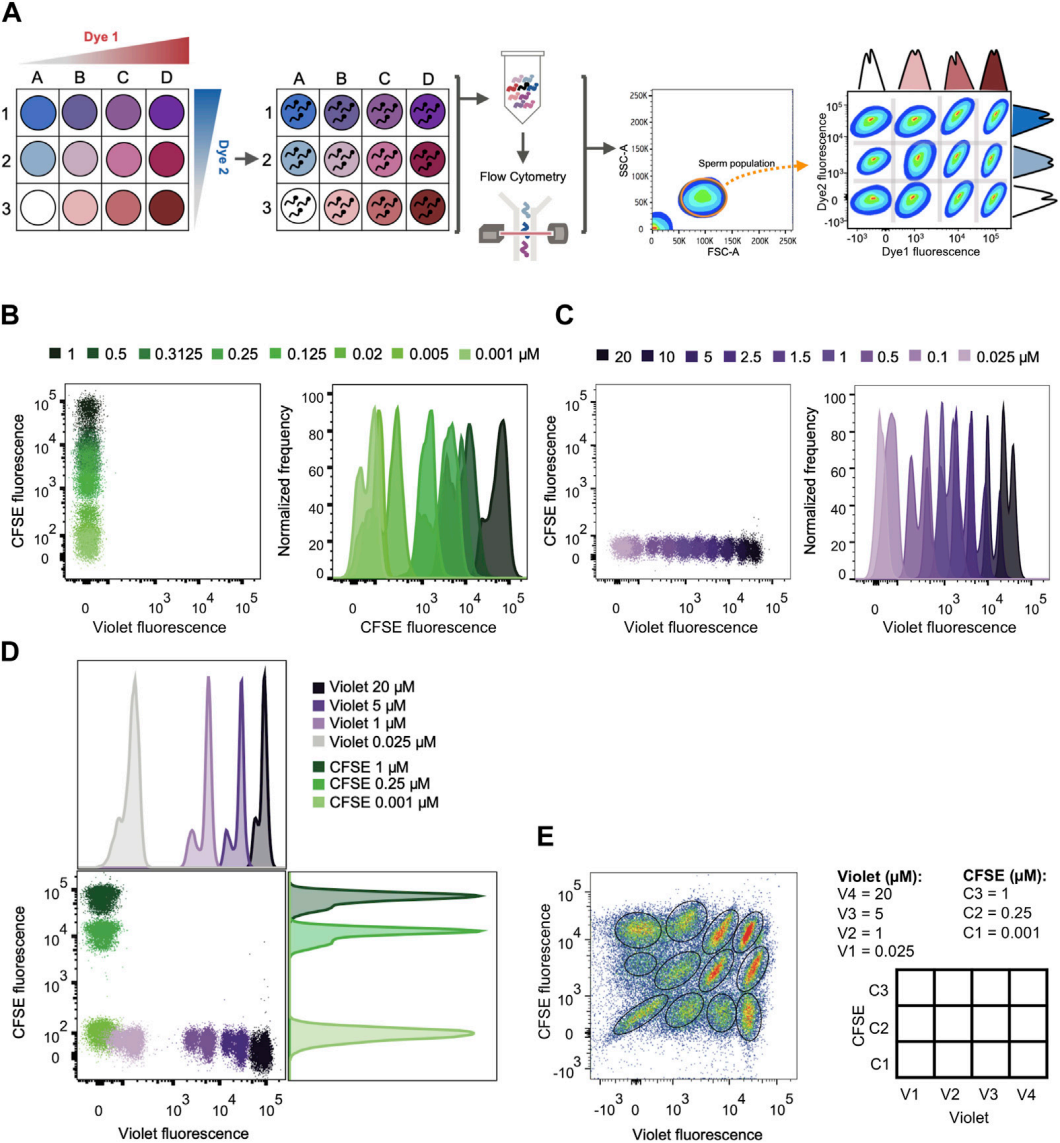
FCB set up in mouse sperm. **(A)** Schematic diagram of the high-throughput screening method: First, each well is given one unique fluorescent signature by the combination of dyes used in different concentrations. Second, sperm samples are distributed in the different wells and loaded with a distinct combination of dyes. After loading, all samples are grouped in a single tube and analyzed by flow cytometry. Following data acquisition, a sperm population is selected from all the events. Each sperm sample is able to be distinguished through their corresponding dye fluorescence signature. **(B)** Representative 2D dot plot of CellTrace™ Violet vs. CellTrace™ CFSE fluorescence (left) and representative histograms of normalized frequency vs. CellTrace™ CFSE fluorescence (right). Sperm were incubated with increasing µM concentrations of CellTrace™ CFSE and its fluorescence raised in a dose dependent manner. **(C)** Representative 2D dot plot of CellTrace™ Violet vs. CellTrace™ CFSE fluorescence (left) and representative histograms of normalized frequency vs. CellTrace™ Violet fluorescence (right). Sperm were incubated with increasing µM concentrations of CellTrace™ Violet and its fluorescence raised in a dose dependent manner. **(D)** Representative 2D dot plot of CellTrace™ Violet (Dye 1) vs. CellTrace™ CFSE (Dye 2) fluorescence, with their corresponding histogram analysis depicting normalized frequency of sperm vs. dye fluorescence. The concentrations selected for CellTrace™ Violet and CellTrace™ CFSE are shown, with no overlapping between each other. **(E)** Representative 2D dot plot of CellTrace™ Violet vs. CellTrace™ CFSE fluorescence of a 4 × 3 matrix, where the population of sperm with each dye combination/signature was defined. The diagram with the dye concentration in each well is depicted on the right as a double-entry table.

Flow cytometry is a high-content, multiparameter platform that allows the analysis of multiple biosignatures at the single-cell level. FCB dramatically reduces reagent consumption, improves flow cytometry experiments throughput, and eliminates staining variabilities between samples (Krutzik and Nolan, 2006). This technique has been widely used in multiple cellular systems (cell lines and primary cell samples) and for different applications in the development of high-throughput screening methods (Krutzik and Nolan, 2006; Krutzik et al., 2008; Clutter et al., 2010; Irish et al., 2010; Stam et al., 2011; Mei et al., 2015). The FCB method is usually used in fixed and permeabilized cells, although FCB of live cells is possible. As detailed in the diagram of Figure 3A, mouse sperm samples were labeled with different intensities of two FCB markers (namely Dye 1 and Dye 2) by incubation with increasing concentrations of these fluorescent dyes. As a result, each sample was labeled with a unique fluorescent signature. Due to their specific characteristics, FCB markers remain covalently attached to the cells, allowing the combination of these multiple sperm samples in a single analysis tube, from which the acquisition by flow cytometry was performed.

For setting FCB, mouse sperm were stained with two dyes that are compatible with the use of propidium iodide (PI) as a viability marker and DiSC_3_(5) as a Em reporter, since their respective spectrum of emission do not overlap and can be easily analyzed by flow cytometry: CellTrace™ CFSE (C34554) and CellTrace™ Violet (C34557). The addition of either dye produced an increase in emission in a concentration-dependent manner (Figures 3B, C). Since the ultimate objective is to identify each sperm sample, 3 concentrations of CellTrace™ CFSE (0.001, 0.25 and 1 µM) were selected in combination with 4 concentrations of CellTrace™ Violet (0.025, 1, 5 and 20 µM), which displayed discrete fluorescent peaks in the histogram (Figure 3D) that can be gated when all the samples were combined (Figure 3E). This combination of dyes allowed us to simultaneously analyze 12 different sperm samples that were clearly identified afterwards (Figure 3E). A maximum concentration of CellTrace™ CFSE 1 µM was used since it was the higher concentration that allowed a proper fluorescence compensation with PI (Supplementary Figure S4).

### High-throughput screening method to identify novel compounds that inhibit CatSper channels

**FIGURE 4:**
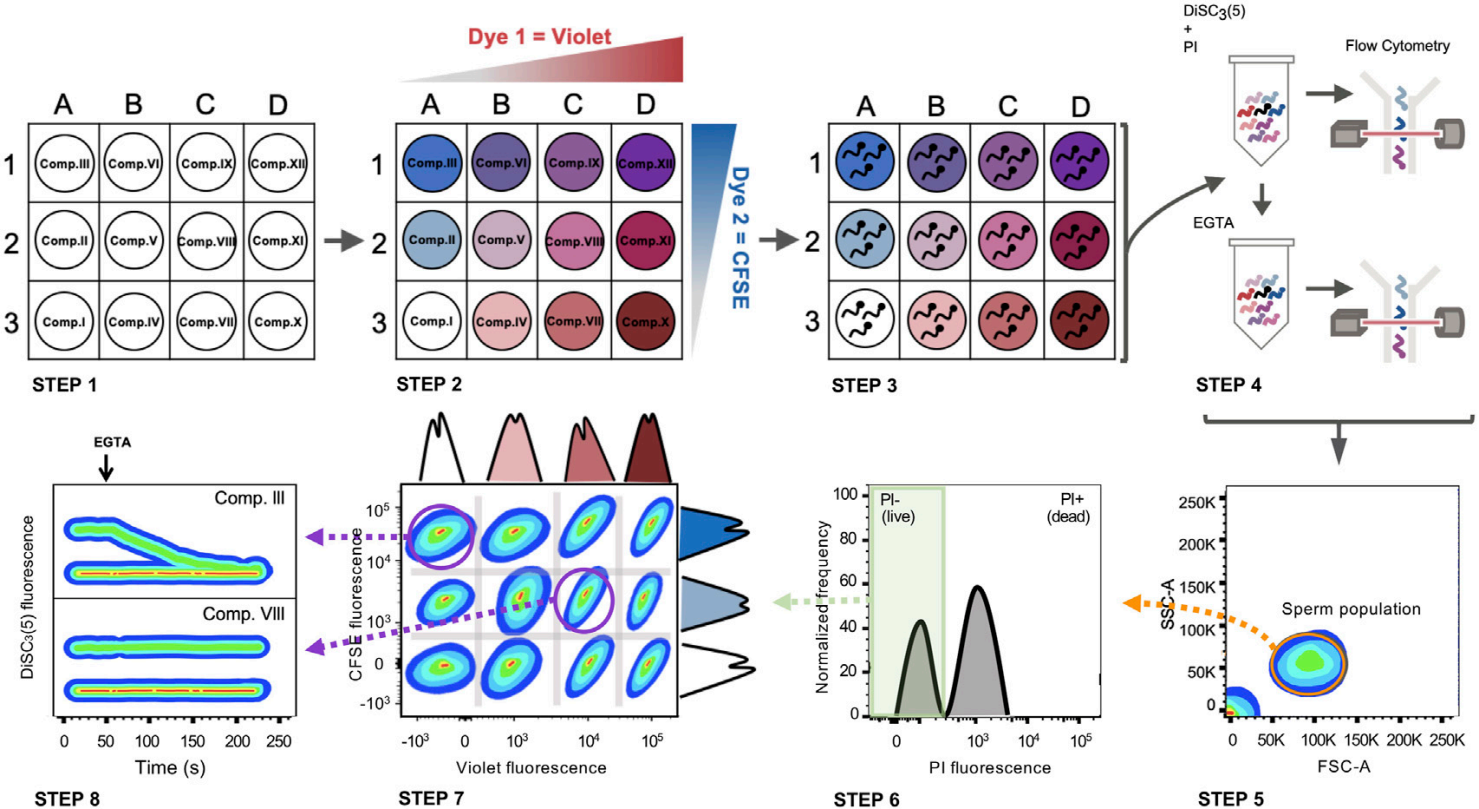
FCB in combination with Em analysis by flow cytometry in mouse sperm. Schematic diagram: STEP 1) A 96 well plate is prepared with a different compound in each well. STEP 2) Each well is loaded with a unique combination of dyes (CellTrace™ Violet and CellTrace™ CFSE). STEP 3) Sperm samples are distributed in different wells and incubated with the compounds while loading with a distinct combination of dyes. STEP 4) After loading, all samples are grouped in a single tube, loaded with DiSC_3_(5) (Em indicator) and PI (viability marker) and analyzed by flow cytometry (basal recording of 30 s). After basal Em acquisition, EGTA 3.5 mM is added and immediately analyzed by flow cytometry again (for 150 s). STEP 5) Following data acquisition, the sperm population is selected from all the events recorded. STEP 6) The live sperm population is selected by gating PI-negative sperm. STEP 7) Live sperm samples can be distinguished through their corresponding dye fluorescence signature. STEP 8) DiSC_3_(5) fluorescence before and after EGTA addition is determined in each population, discriminating if there is any compound that promotes CatSper closure. Comp. III = does not inhibit CatSper. Comp. VIII = CatSper inhibitor.

To develop a high-throughput method to screen drugs with the capacity to block CatSper channels, FCB flow cytometry in combination with the potentiometric probe DiSC_3_(5) as a reporter of Em was employed. The sequence of procedures is summarized in Figure 4, where the process was divided into 8 steps as follows:

Step 1: Dilutions for each compound were performed in the appropriate medium as previously described in the Mat&Met section.

Step 2: Each well was labeled with unique signatures or “barcodes” of fluorescent dyes. Two different dyes were used to produce 12 barcodes: CellTrace™ CFSE (C34554) and CellTrace™ Violet (C34557), as previously described in the Mat&Met section.

Step 3: The appropriate concentration of mouse sperm (6–9 × 10^6^/ml final concentration per well) in NC TYH medium was added to each well and incubated to allow both compounds and dyes to be simultaneously incorporated into sperm. The omission of BSA is critical in this step since this protein may bind to these compounds decreasing the effective concentration in the well. Each 12-well matrix also included one positive and one negative control. As positive control (CatSper inhibition), sperm were incubated in the presence of 10 µM HC-056456. As a negative control (normal CatSper activity), sperm were incubated in the presence of the vehicle used to dissolve the drugs (either DMSO or water).

Step 4: After incubation, NC TYH medium was added to each well and the content of one 12-well matrix was combined into one tube containing additional NC TYH medium. Sperm were washed by centrifugation to remove the excess of unbound dyes. Then, sperm samples were resuspended in NC TYH medium and the Em-sensitive probe DiSC_3_(5) was added. At this point, PI was added to differentiate between live and dead sperm. The tube was further analyzed by flow cytometry to acquire basal Em, and after EGTA 3.5 mM addition to promote the depolarizing influx of Na^+^ through CatSper, acquisition continued for 150 additional seconds.

Step 5: The analysis of the FCB experiment was performed with FlowJo software. In the FSC-A vs. SSC-A dot plot, the sperm population was selected.

Step 6: Those cells that uptake PI (dead sperm) were gated out and the following analysis was only performed with the population of live sperm (PI negative).

Step 7: During software analysis the samples were distinguishable based on their fluorescence intensity in each FCB marker channel. Sperm within each well possess a unique fluorescent signature. Using the FlowJo software, plotting the corresponding channel for Dye 1 (CellTrace™ Violet) vs. the corresponding channel for Dye 2 (CellTrace™ CFSE) revealed distinct populations that correspond to the original wells that were barcoded.

Step 8: Once gated, the populations were analyzed according to DiSC_3_(5) fluorescence before and after the EGTA addition. As an example, two different conditions are displayed. On the bottom, a situation where a given compound inhibits CatSper function is displayed, whereas on top, a representative example where a given compound is not blocking CatSper is shown.

**FIGURE 5:**
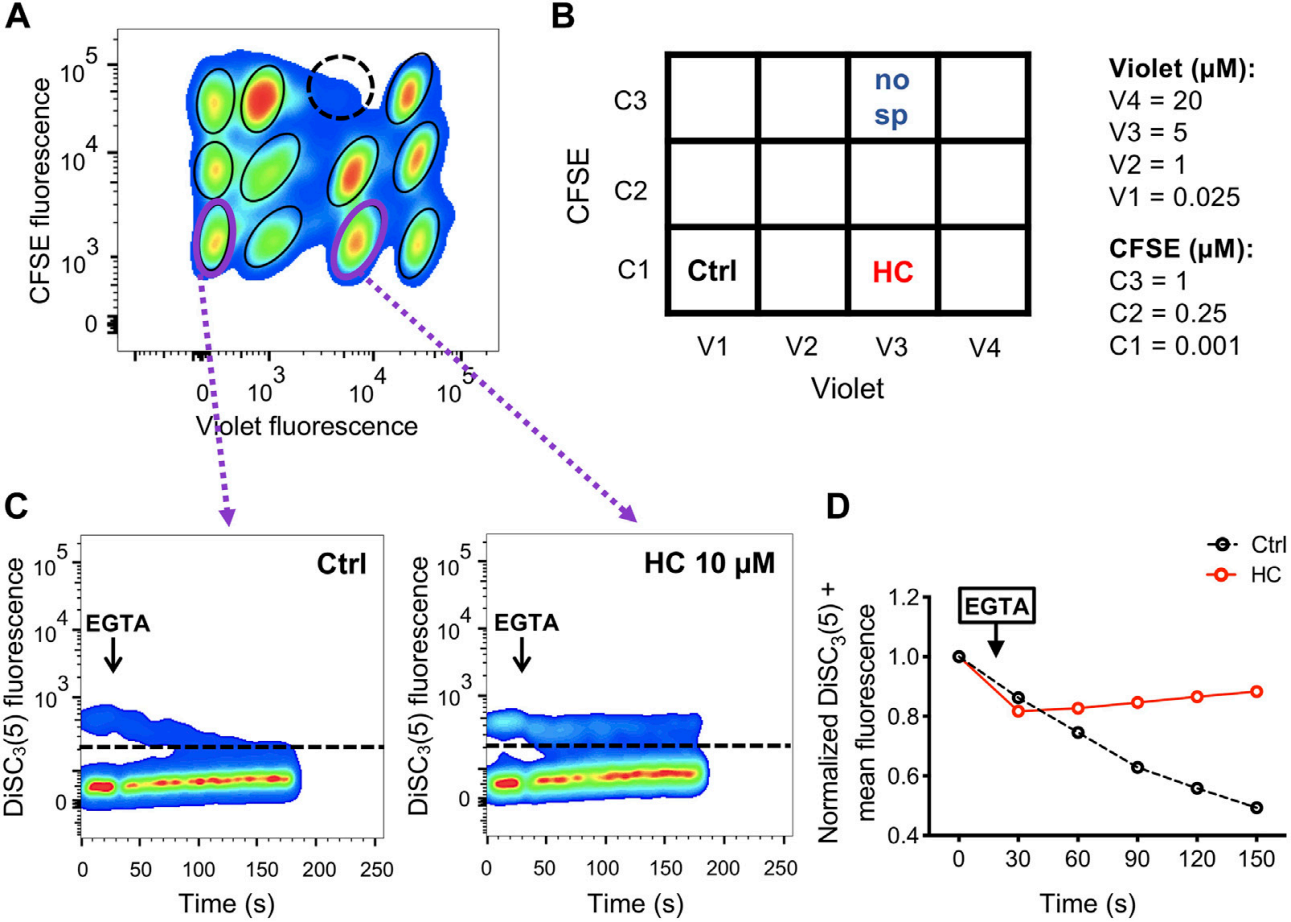
**(A)** Representative 2D dot plot of CellTrace™ Violet vs. CellTrace™ CFSE fluorescence of a 4 × 3 matrix, where the population of sperm with each dye combination/signature was defined. As an additional control of proper identification in the matrix, in one well sperm were not added (dashed-line circle). **(B)** Diagram showing the arrangement of compounds (HC: CatSper inhibitor; Ctrl: NC + DMSO vehicle; no sp: no sperm were added) within the 4 × 3 matrix. The dye concentration in each well is depicted as a double-entry table. **(C)** Representative 2D dot plot of DiSC_3_(5) fluorescence vs. time (seconds) analysis for the positive (HC) and negative (Ctrl) controls. The addition of 3.5 mM EGTA (black arrow) induced a decrease in the population of sperm with high DiSC_3_(5) fluorescence due to Em depolarization in the NC control condition (with only vehicle) but not in sperm that were incubated with 10 µM HC-056456. The population of sperm that responds by decreasing the Em was established in the NC control condition and extrapolated to the other conditions (dashed line). Sperm cells above the dashed line [higher DiSC_3_(5) fluorescence] were arbitrarily identified as DiSC_3_(5) +. **(D)** DiSC_3_(5) fluorescence mean was determined in the sperm population with higher DiSC_3_(5) fluorescence: DiSC_3_(5) + [dashed line in **(C)**] before (time = 0 s) and after (black arrow) addition of 3.5 mM EGTA. In each condition, the fluorescence mean was normalized to the basal fluorescence (0 s).

**FIGURE 6:**
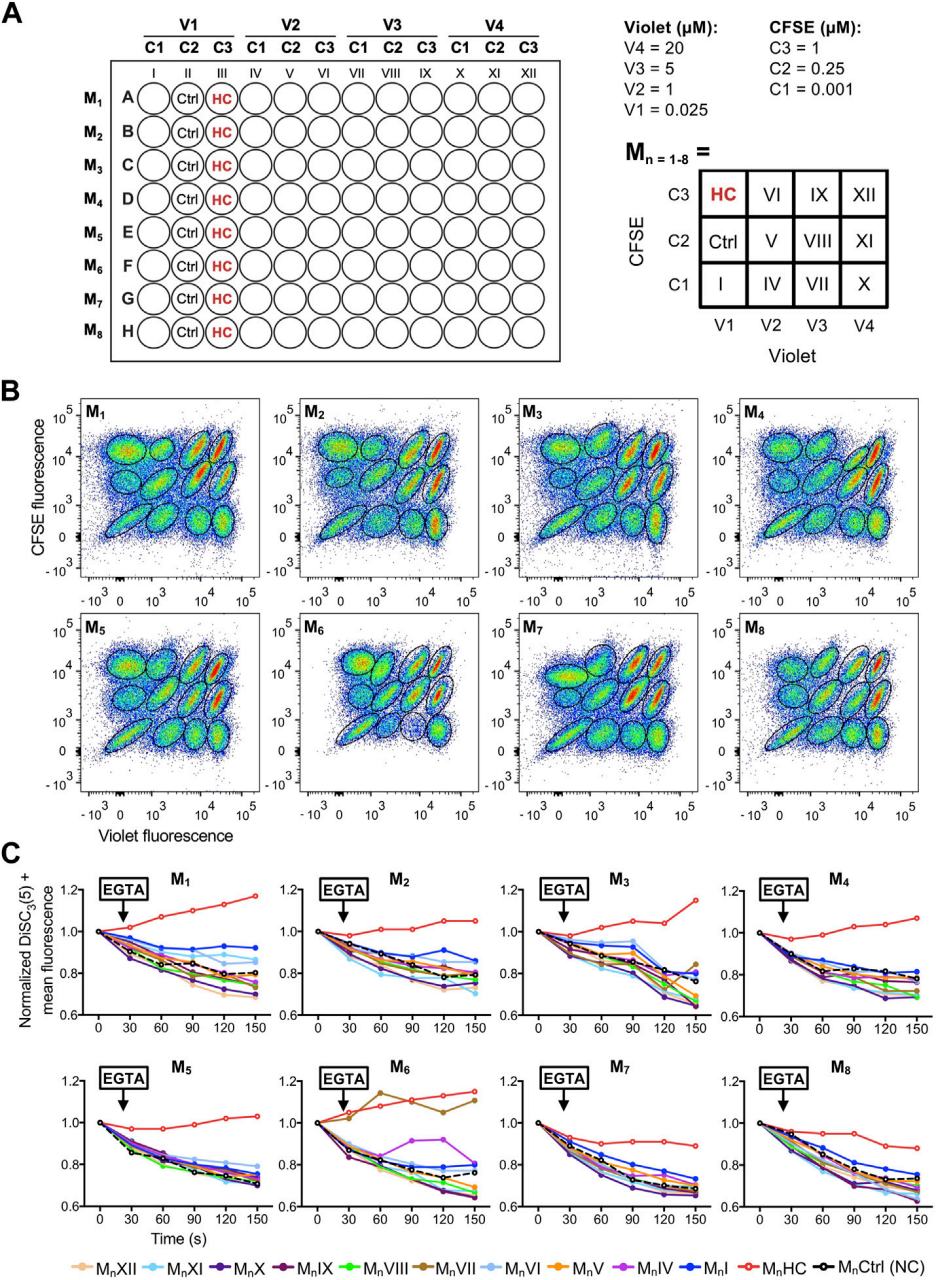
**(A)** Left-panel: schematic diagram of a 96-well plate showing the arrangement of compounds (all tested at 10 µM), dyes (V1-4: CellTrace™ Violet concentrations; C1-3: CellTrace™ CFSE concentrations), and controls (Ctrl: NC + DMSO vehicle; HC: 10 µM HC-056456). Each row corresponds to a different matrix (M_n=1–8_). Right-panel: schematic diagram of the compound arrangement within each 4 × 3 matrix. The dye concentration in each location is depicted as a double-entry table. **(B)** Representative 2D dot plots of CellTrace™ Violet vs. CellTrace™ CFSE fluorescence of the eight 4 × 3 matrixes from the 96-well plate, where the population of sperm with each dye combination/signature was defined. **(C)** DiSC_3_(5) fluorescence mean was determined in DiSC_3_(5) + population before (time = 0 s) and after (black arrow) addition of 3.5 mM EGTA. In each condition, the fluorescence mean was normalized to the basal fluorescence (0 s). Each color represents a different compound (M_n_I-XII; *n* = 1–8) obtained through its corresponding dye fluorescence signature.

Figure 5A shows an example of a single 4 × 3 matrix where different sperm populations can be distinguished depending on their barcoding dyes concentration. The position of a given treatment within the matrix is diagrammed in Figure 5B as a double-entry table, where it can be defined through its corresponding CellTrace™ Violet—CellTrace™ CFSE signature. For example, in V3-C3 position no sperm were added as a control to corroborate proper sperm sample identification (Figure 5A, dashed-line circle). In V3-C1, a well-known CatSper inhibitor (10 µM HC-056456) was added as a positive control, while as a negative control only DMSO (vehicle) was added in position V1-C1. After selecting the appropriate population, distinguished by its unique signature provided by the FCB (which corresponds to a specific position of the initial microplate), DiSC_3_(5) fluorescence was monitored over time (Figure 5C). The mean DiSC_3_(5) fluorescence of the hyperpolarized subset of cells was quantified and displayed in Figure 5D. As observed, the presence of the CatSper inhibitor blocked the EGTA-induced depolarization (Figures 5C, D, HC). In contrast, addition of DMSO (Figures 5C, D, Ctrl) did not inhibit CatSper and displayed a robust depolarization. By using a 96-well microplate, eight 4 × 3 matrices could be included. If one positive control (HC-056456 or CatSper1 KO sperm) and one negative control (NC + DMSO) are used per matrix, a total of 80 compounds could be tested in 40 min. A representative example of this procedure is presented in Figure 6, where 80 compounds from a FDA-Approved Drug Library were tested (Supplementary Table S1). To assess intra-experiment variability, the mean of the DiSC_3_(5) + fluorescence from the eight NC and HC controls was calculated and displayed in Supplementary Figure S5.

## Discussion

It is currently estimated that one in four pregnancies is unplanned worldwide (Shah and Åhman, 2010; Bearak et al., 2018), based on studies that analyzed data bases from 1990–2014 in more than 100 countries. In addition, available male contraceptive methods are scarce, resulting in a gender imbalance in decision-making related to family planning. Nowadays, most men confirm that they would be interested in using new fertility control methods, if available (Heinemann et al., 2005), while many women would be willing to rely on their partner on this matter (Glasier, 2010). Initially, most efforts were directed to the development of male hormonal contraceptives, resembling the female birth control pill. However due to several side effects associated with hormonal treatments none have reached the market, and the acceptance among users is low. More recently, research has been focused on the discovery of non-hormonal targets, particularly compounds directed to block sperm production on the testis, sperm maturation on the epididymis, and/or sperm function (Long et al., 2021). A male contraceptive must be specific, effective, safe, reversible, and accessible. Taking this into consideration CatSper emerges as an attractive male contraceptive target since it is a sperm-specific Ca^2+^ channel, only localized in the flagellum, and essential for sperm fertilizing ability. Targeted disruption of each of the four murine CatSper ion channel proteins was shown to lead to infertility. Similarly, humans with CatSper loss-of-function mutations have shown male infertility due to hyperactivation impairment, without systemic effects (Avidan et al., 2003; Avenarius et al., 2009; Smith et al., 2013). Therefore, a pharmacological CatSper inhibitor might be used as an on-demand male contraceptive and/or as a female treatment to prevent sperm migration through the oviduct.

It is well established that CatSper regulation is promiscuous since it can be non-specifically inhibited or activated by numerous compounds (Rennhack et al., 2018; Rahban et al., 2021). This channel’s intrinsic feature makes it challenging to discover specific candidates to inhibit its function. For example, some of the pharmacological CatSper inhibitors available nowadays are not specific, such as Mibefradil (Carlson et al., 2009) and NNC 55-0396 (Huang et al., 2004), which are T- and L-type Ca^2+^ channel blockers. CatSper inactivation with HC-056456 (Carlson et al., 2009) was recently investigated showing that intrauterine insemination of treated sperm resulted in a reduced percentage of fertilized oocytes (Curci et al., 2021), but further *in vivo* experiments are required. Others such as RU 1968 (Rennhack et al., 2018), have not been well characterized and tested *in vivo*.

In addition, the high structural complexity of CatSper has impeded its heterologous expression making *in vitro* studies difficult. In this regard, patch clamp electrophysiology is the gold standard technique for direct measurement of channel ionic currents. Still, conventional manual patch clamp requires highly skilled personnel, it is time-consuming and therefore has low throughput. Recently, the advent of automated patch clamp technologies enabled high-throughput direct measurement of ion channel function (Mathes, 2006; Picones et al., 2016). Although this technique has been applied to a wide variety of cell types, it presents additional challenges when it comes to sperm, a very tiny cell with scarce cytoplasm and constant movement. One way around this has been the use of high-throughput assays with alternative measures of CatSper function such as [Ca^2+^]_i_ (Carlson et al., 2022). Although informative, this parameter does not directly assess CatSper channel function and has key limitations, as several compounds can generate changes in [Ca^2+^]_i_ without significant effects on CatSper.

Data presented in this paper have also validated the indirect assessment of CatSper function through Em determination. This approach has been used in previous reports from our group (Torres-Flores et al., 2011; Ernesto et al., 2015; Ritagliati et al., 2018; Stival et al., 2018; Luque et al., 2021) to determine the inhibitory effect of proteins or compounds. Possible and valid concerns about this method are related to the existence of other ion channels that may also become permeable to monovalent ions when Ca^2+^ is chelated. Now, we fully validated this functional assay using CatSper1 KO mice. The depolarizing Na^+^ influx occurs mainly through CatSper channels since many sperm remained hyperpolarized in CatSper1 KO. This observation suggests that, although we cannot rule out the contribution of other channels, it may not be significant in magnitude to alter the results obtained by this methodology. Thus, Em determination before and after EGTA addition (Em assay) is a reliable, simple, reproducible, and robust functional assay for measuring indirectly the CatSper opening extent, by both spectrofluorometry and flow cytometry. It is essential to consider that there might be compounds with the ability to change basal sperm Em through the blockage of other channels or electrogenic exchangers, which could compromise the sensitivity of the test.

FBC combined with the Em assay provides a new high-throughput tool for discovering novel CatSper blockers. This phenotypic assay presents future challenges such as hit validation that require follow-up testing (Moffat et al., 2017). In addition, FBC in live sperm itself emerges as a limitless and therefore valuable tool. Considering only the proper combination of fluorophores, this system could be applied in the future to determine novel blockers of acrosome reaction (by using for example the mouse model with EGFP in their acrosomes (Hasuwa et al., 2010) or FM4-64 when the desired extension to humans is performed (Balestrini et al., 2021)), intracellular [Ca^2+^]_i_ increase (Fluo-4, Fluo-3 or Fura), intracellular pH alkalinization (BCECF), as well as other biochemical and/or functional events associated with sperm capacitation.

## Data Availability

The original contributions presented in the study are included in the article/Supplementary Material, further inquiries can be directed to the corresponding authors.

## Supplementary material

SUPPLEMENTARY FIGURE S1 Difference between depositing the sperm in the corresponding well containing the dye from above without submerging the tip (BEFORE) and submerging the tip (AFTER). The sperm population results in a more homogeneous loading when submerging the tip. Representative 2D dot plot of CellTrace™ Violet vs. CellTrace™ CFSE fluorescence, with their corresponding histogram analysis depicting the normalized frequency of sperm vs. dye fluorescence. **(A)** Sperm sample loaded with CellTrace™ CFSE 1 µM. **(B)** Sperm sample loaded with CellTrace™ Violet 20 µM.

SUPPLEMENTARY FIGURE S2 The Em assay was evaluated in two different mouse strains: A) C57BL/6 and B) Hybrid F1 (C57BL/6 male × BALB/c female) mature (10–12 weeks-old) male mice were used. Representative 2D dot plots of DiSC_3_(5) fluorescence vs. time (seconds) analysis are shown. The addition of 3.5 mM EGTA (black arrow) provokes a decrease in the population of sperm with high DiSC_3_(5) fluorescence due to Em depolarization in the control condition (DMSO vehicle; left), but not in the presence of 10 µM HC-056456 (right).

SUPPLEMENTARY FIGURE S3 CatSper activity was assessed by measuring Em in individual live cells by flow cytometry. **(A)** Representative 2D dot plot of DiSC_3_(5) fluorescence vs. time (seconds) analysis. The addition of 3.5 mM EGTA (black arrow) provokes a decrease in the population of sperm with high DiSC_3_(5) fluorescence due to Em depolarization in the control condition (DMSO vehicle). The addition of the specific CatSper inhibitor HC-056456 prevents this decay in a concentration-dependent manner, where at 10 µM the maximum effect is reached. **(B)** Quantification of at least 3 independent experiments (mean ± SEM) is shown. The population of sperm that responds by decreasing the Em was established in the control condition and extrapolated to the other conditions [**(A)**, dashed line]. DiSC_3_(5) fluorescence mean was determined in DiSC_3_(5) + population before (time = 0 s) and after (black arrow) addition of 3.5 mM EGTA. In each condition, the fluorescence mean was normalized to the basal fluorescence (0 s). No differences were found between HC 10 µM vs. time matched HC 20 µM. Both, HC 10 µM and HC 20 µM were significantly different vs. time matched control condition (DMSO). Two-way ANOVA with repeated-measures in combination with Tukey’s multiple comparison test was performed.

SUPPLEMENTARY FIGURE S4 CellTrace™ CFSE has some emission overlap with PI. Representative 2D dot plot of CellTrace™ CFSE vs. PI fluorescence before and after compensation. Increasing concentrations of CellTrace™ CFSE are shown. The higher concentration of CellTrace™ CFSE that allows optimal discrimination of populations in combination with PI is 1 µM.

SUPPLEMENTARY FIGURE S5 Quantification of the eight NC and HC controls used in the 96-well plate to evaluate intra-experiment variability. DiSC_3_(5) fluorescence mean was determined in DiSC_3_(5) + population before (time = 0 s) and after (black arrow) addition of 3.5 mM EGTA. In each condition, the fluorescence mean was normalized to the basal fluorescence (0 s). Mean ± SEM is shown.

SUPPLEMENTARY TABLE S1 List of the 80 compounds from the FDA-Approved Drug Library used in the representative screening-experiment shown in Figure 6.
