# Supplementary figures and images for: High-throughput screening method for discovering CatSper inhibitors using membrane depolarization caused by external calcium chelation and fluorescent cell barcoding
Source: Front Cell Dev Biol. 2023 Jan 19;11:1010306. doi: 10.3389/fcell.2023.1010306 (PMC9892719; doi:10.3389/fcell.2023.1010306)

A

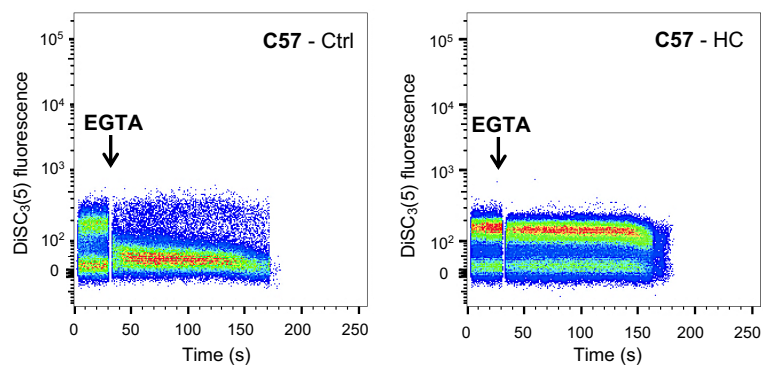

B

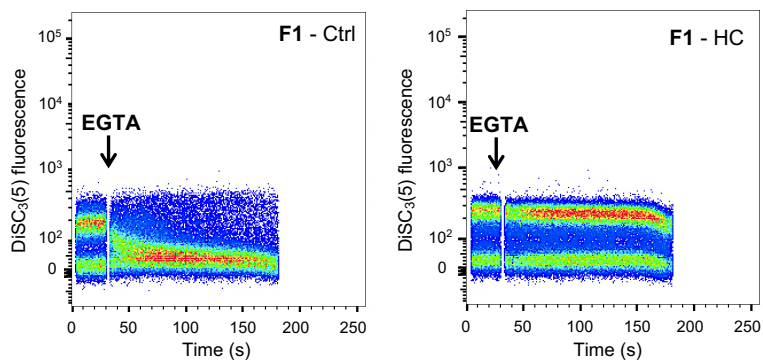

Supplement: Supplementary file 1 [file DataSheet2.PDF]

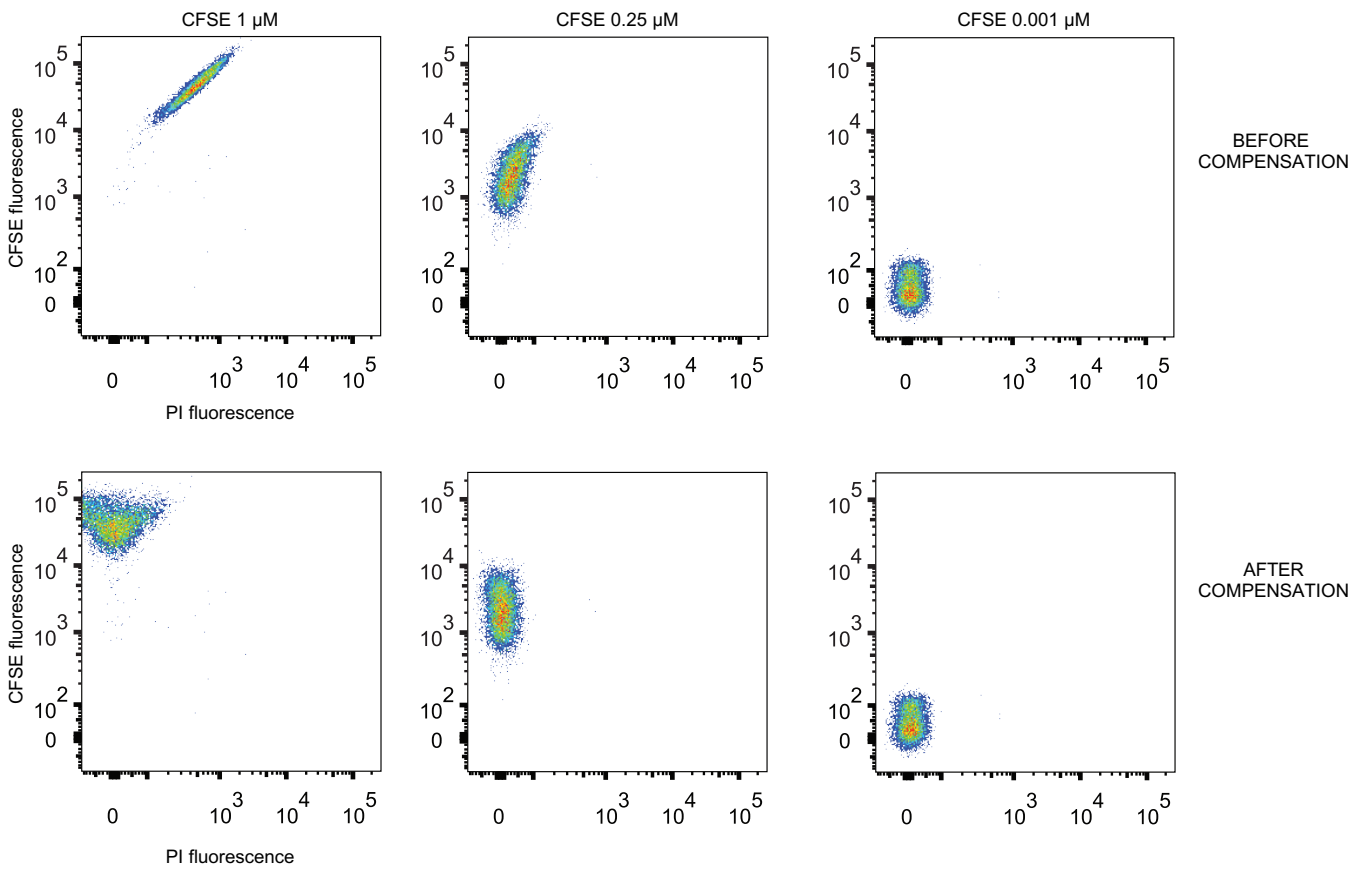

Supp. Fig.4

Supplement: Supplementary file 2 [file DataSheet4.PDF]

A

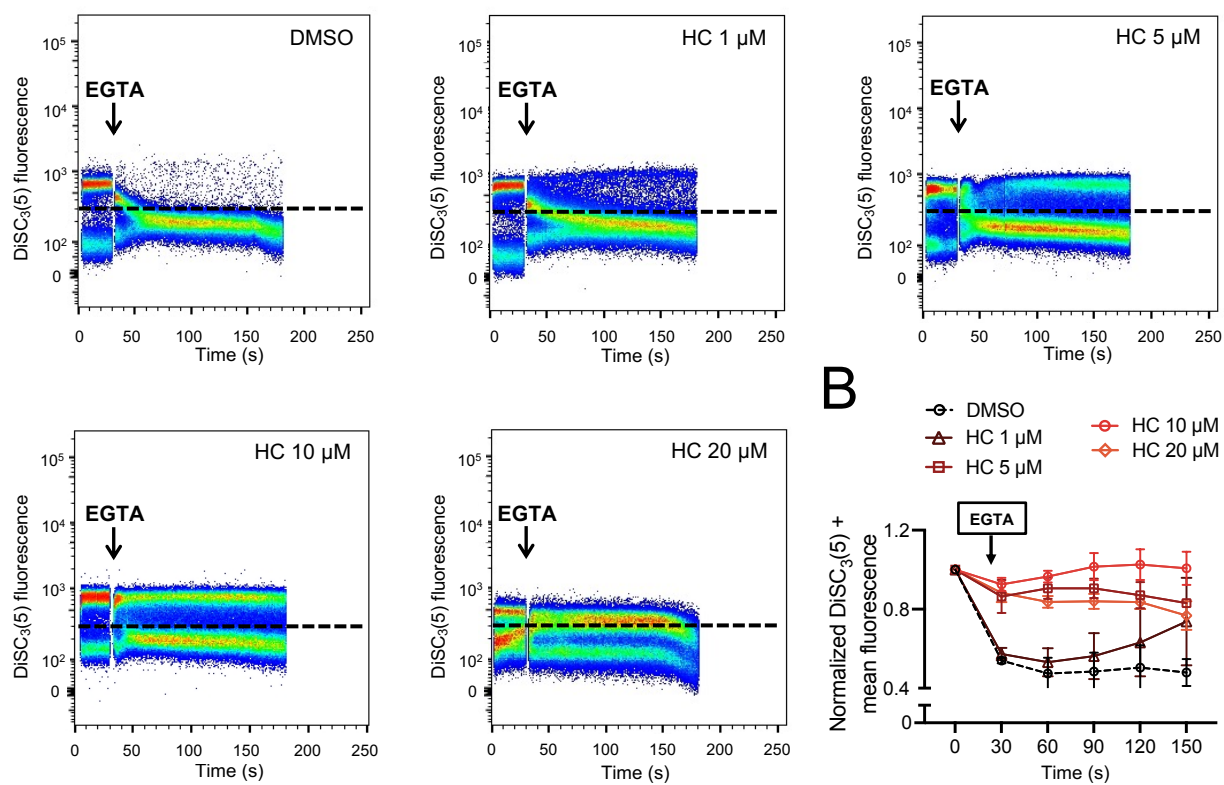

B

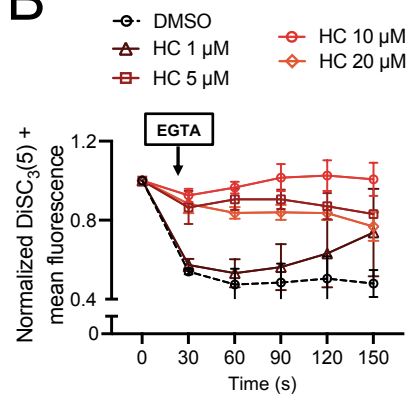

Supplement: Supplementary file 3 [file DataSheet3.PDF]

A

CFSE 1  $\mu$ M

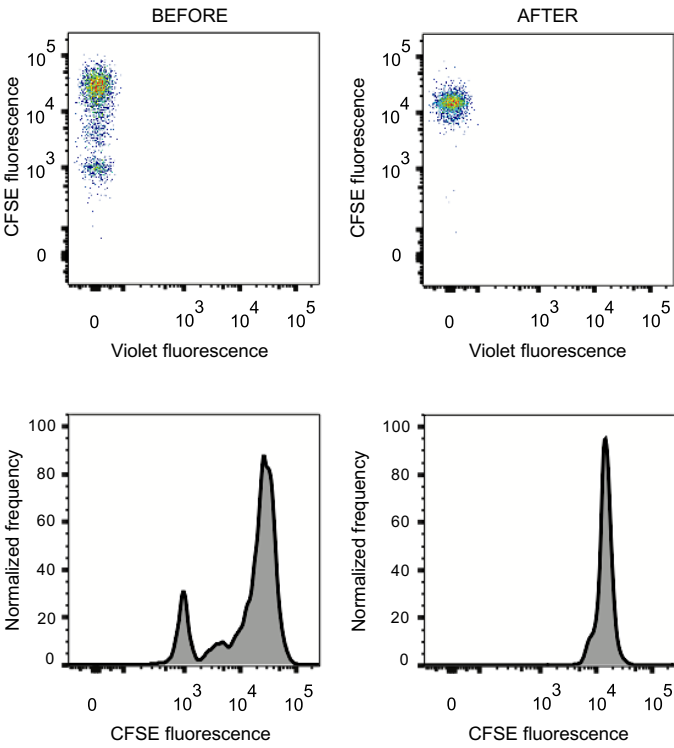

B

Violet 20  $\mu$ M

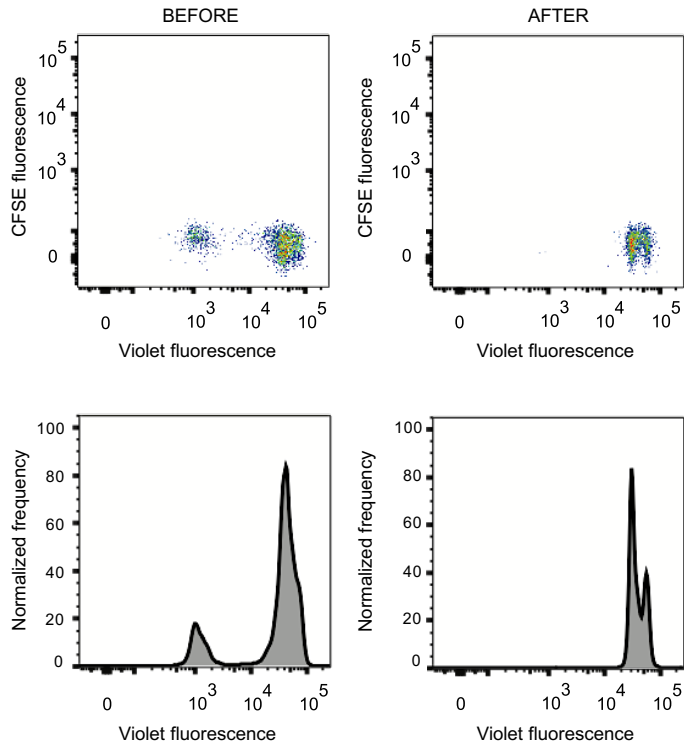

Supplement: Supplementary file 4 [file DataSheet1.PDF]

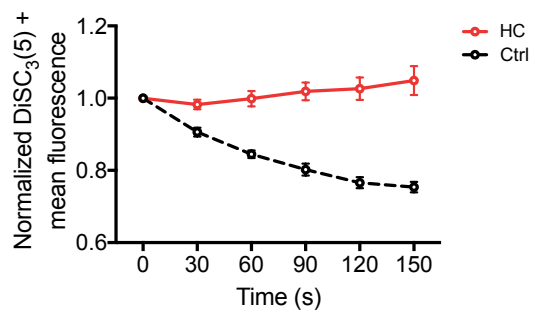

Supplement: Supplementary file 5 [file DataSheet5.PDF]
